# Structural Relationships between Learning Emotion and Knowledge Organization and Management Processes in Distance Learning Environments: “An Applied Study”

**DOI:** 10.3390/ejihpe13090114

**Published:** 2023-08-24

**Authors:** Shoeb Saleh, Rommel AlAli, Yousef Wardat, Mohammed Al-Qahtani, Yasser Soliman, Mamdouh Helali

**Affiliations:** 1 The National Research Center for Giftedness and Creativity, King Faisal University, Al-Ahsa 31982, Saudi Arabia; sgsaleh@kfu.edu.sa (S.S.); msqahtani@kfu.edu.sa (M.A.-Q.); mhelali@kfu.edu.sa (M.H.); 2 Department of Educational Technology, Faculty of Education, Sohag University, Sohag 82524, Egypt; 3 Higher Colleges of Technology, Al Ain 15551, United Arab Emirates; 4 Department of Educational Leadership, Faculty of Education, King Faisal University, Al-Ahsa 31982, Saudi Arabia; 5 Applied College, King Faisal University, Al-Ahsa 31982, Saudi Arabia; ymsoliman@kfu.edu.sa

**Keywords:** distance learning, harmonious passion emotional obsession, self-regulation, knowledge management

## Abstract

The widespread adoption and expansion of distance learning necessitates the consideration of issues related to learning passion, which is the strong desire of learners towards a specific activity of high value and meaning that enables the use of relevant cognitive and behavioral strategies to acquire, store, apply, share, use, absorb, and create knowledge. The current study aimed to measure learners’ practices of learning emotion processes, knowledge management, and organization in distance learning environments using tangible indicators. The study utilized a descriptive correlational approach to identify the effects of the study variables, including learning emotion (harmonious passion—emotional obsession), on knowledge management through the mediating role of self-regulation in distance learning environments. The results show that learners’ practices of learning emotion processes, knowledge management, and organization in distance learning environments were higher than average, and there was a significant correlation between emotion, self-regulation, and knowledge management. Additionally, learning emotion (harmonious passion and emotional obsession) had a statistically significant effect on self-organization processes and knowledge management. Structural equation modeling analysis confirmed the validity of the proposed path model, indicating that self-regulation plays a crucial role in mediating the relationship between emotion and knowledge management in distance learning environments.

## 1. Introduction

The advent of the distance education system was a direct response to the challenges brought forth by the information and knowledge revolution in the modern era. To overcome the constraints of time and location, contemporary techniques and tools such as e-learning and digital technology are being employed in university education for conveying information to students. The effectiveness of this approach is contingent upon the design of the technological educational environment and the consideration given to its fundamental components. Additionally, a multitude of studies have emphasized the urgent need for interactive learning environments that actively engage students, foster their motivation to persist in learning, and augment their ability to apply the acquired knowledge in real-life situations [1,2].

With the onset of the COVID-19 pandemic, attention turned into a search for ways that make the learner a vital and effective component and that enables them to think about how to deal with educational situations, and to find several solutions and organize and evaluate them in order to reach the right solution, so the characteristics of this stage require individuals who have the ability of continuous self-learning. Additionally, this does not happen unless the individual has an internal motive imposed by the technological educational environment that urges him to learn constantly, through what modern, visual, motor and motor technology provides, which is necessary in the learning process [1].

The progress of technology has significantly elevated the quality of education by establishing an interactive and immersive learning environment tailored to individualized preferences [3]. As a result, the functions related to managing knowledge (KM) have gained increasing significance and have become essential for effective learning in the digital age. These functions encompass skills such as acquiring and storing knowledge, applying knowledge, sharing knowledge, and forming knowledge [4,5]. Songhao et al. [6] have proposed that individuals residing in a technology-driven society should possess the capability to gather, amass, distribute, and utilize knowledge through diverse educational materials [6]. Additionally, research has confirmed that knowledge management competencies are positively associated with technological innovation, including both product and process innovation. Therefore, comprehending the factors that influence knowledge management within the e-learning community is crucial for educating the younger generation [7].

Self-regulation pertains to a process that assists learners in constructing their own learning activities through the utilization of relevant cognitive and behavioral approaches [8]. Strategies for self-regulated learning encompass techniques designed for acquiring knowledge, including its organization and transformation, maintaining records and control, and structuring the learning environment [9,10]. It is important to note that a correlation exists between self-regulation and knowledge management, as learners who consistently employ self-regulatory processes utilize strategies aimed at gaining knowledge or skills to achieve a higher level of success [11]. Moreover, numerous empirical research studies have indicated that individuals who embrace self-regulatory processes in their learning demonstrate improved knowledge acquisition and the capacity to develop a more intricate knowledge network [12,13,14].

Motivation holds significant importance in the realm of learning. Traditionally, scholars have categorized motivation into extrinsic and intrinsic forms. However, a more comprehensive concept named “emotion”, based on distinct psychological mechanisms of internalization, has been introduced by psychologists in recent times [15,16]. This notion defines “passion” as an inclination or desire for an activity that involves time and energy investment. It suggests that varying internalization approaches yield different types of emotion. This understanding has led to the development of a dual emotion model comprising “harmonious emotion” and “obsessive emotion”, offering insights into the cognitive processes of motivation within specific learning contexts.

While some researchers have highlighted the significance of passion for knowledge management abilities [17,18], limited empirical investigations have explored how these two emotions correlate with knowledge in e-learning environments. Given the rapid expansion of hypermedia platforms, scholars have also directed their attention towards learners’ self-regulation within the context of e-learning [19,20]. Certain studies have suggested that learners in novel learning environments, like e-learning, necessitate proactive attitudes and strategies to effectively construct knowledge [19,21,22].

Individuals with adept self-regulation skills tend to engage actively in the learning process through their conduct, motivation, and metacognition [23]. Consequently, it is hypothesized that proficient self-regulatory learners are better positioned to apply their knowledge management proficiencies for effective self-directed learning.

### 1.1. Problem Study

In various periods, distance learning issues have primarily focused on the challenges related to preparing and designing digital content, emphasizing technical infrastructure, and all other aspects encompassing the operations of the distance learning system. Numerous studies and research endeavors have addressed the most critical pillars of the distance learning system, such as establishing goals, constructing digital content, developing learning scenarios, identifying mechanisms and tools for evaluating diverse learning outcomes, and others. However, the focus on the recipient of this service has been relatively limited. Specifically, how can learners comprehend the designed content? How is the prepared content and learning material organized? How can learners relate the digital content to their personal experiences? How can learners manage their time and organize themselves for learning? How can learners manage their knowledge in e-learning environments? These questions represent a set of factors that significantly influence the success of the distance learning system, which is closely linked to the individual’s learning methods in this electronic environment.

Notwithstanding the significance of the distance learning system as a means for enhancing learners’ capacity to achieve their utmost potential, it presents numerous challenges. These challenges encompass the imperative of attaining favorable outcomes that duly consider the learner’s personality development and behavior, while also accounting for the distinct characteristics inherent in this non-conventional mode of learning. Moreover, the system demands robust standards that ensure the maintenance of educational quality [24]. Despite the fact that distance learning systems are founded on various factors, the most crucial of which is the motivation and passion for metacognitive research of learners, little attention has been paid to comprehending the mechanisms through which different types of emotion influence knowledge management in distance learning environments [25]. Given that emotion represents the analogous and most complementary description of motivation based on different psychological mechanisms of assimilation, it is crucial to identify the role played by motivation and passion in providing learners with the skills to organize and manage knowledge. Therefore, the present study aims to investigate the different effects of learning emotion (harmonious passion and obsessive passion) on knowledge management, mediated by self-regulation in distance learning environments, utilizing structural equation modeling.

Consequently, the primary aim of the present study is to address the following research questions:

1. What is the degree of students’ practice of learning emotion, knowledge management, and organization in distance learning environments?
2. What is the statistical significance at level (0.05) of students’ responses to scales of emotion, self-regulation, and knowledge management concerning gender—specialization—level of use of computers—acquired computer courses?
3. What is the relationship between learning emotion (harmonious passion—emotional obsession) and self-regulation and knowledge management in distance learning environments among students?
4. What is the effect of learning emotion (harmonious passion—and emotional obsession) on students’ self-regulation and knowledge management in distance learning environments?

Furthermore, the current study seeks to test a main hypothesis: “Harmonious passion and obsessive passion significantly contribute to explaining the individual differences in knowledge management, mediated by self-regulation, in distance learning environments for students”.

### 1.2. The Importance of the Study

The significance of this study lies in the domain of organizing digital content and investigating the behavioral practices of learners in distance learning environments, along with the factors influencing them. The findings of the study are expected to be beneficial in guiding researchers to develop training programs for learners that are based on an understanding of the learning emotion processes and their role in enhancing the organization and management of knowledge in electronic learning environments.

## 2. Literature Review

Numerous prior studies and literature have examined the topics of motivation and passion for research in e-learning. In this regard, the literature relevant to the present study can be categorized into three main sections:

### 2.1. Studies That Have Examined the Reciprocal Influences of Passion and E-Learning

In the same vein, prior research has explored the relationship between e-learning and academic passion among students. For instance, Hussein’s [26] study found a positive correlation between e-learning and academic passion [26]. Similarly, Youssef’s [27] study revealed that the pattern of reinforcement (immediate/intermittent) in a mini e-learning environment had a significant impact on academic achievement, passion, and reduced mental wandering among low and high self-efficacy education technology students [27]. Soares’ [28] study indicated that e-learning models had a high impact on the development of scientific passion and digital culture [28]. Additionally, Brik and Al-Jariwi’s study concluded that there is a statistically significant correlation between psychological distance in its various dimensions and the total degree of academic passion [29]. Furthermore, Greenberger’s study [30] examined the relationship between academic passion (consensual and compulsive) and face-to-face or online teaching and found that 95% of the study sample who studied online were enthusiastic about e-learning and had academic passion for online learning [30].

### 2.2. Studies That Have Focused on Self-Regulation and Knowledge Management in Distance Learning Environments

Yeh’s [5] study aimed to investigate whether meaning-making, self-regulation, and knowledge management (KM) competencies would interact with a 17-week KM-based treatment and influence creativity in e-learning. The results showed that meaning-making indirectly affected creativity through knowledge management, and self-regulation affected creativity directly and indirectly through knowledge management. Additionally, college students with a higher level of knowledge and ability to self-regulate benefited more from the training than their peers [5]. Similarly, Hu and Driscoll [19] conducted a study to examine the effects of self-regulated learning (SRL) strategy training on learners’ achievement, motivation, and use of the strategy in an enhanced web-based college success course. The results indicated that the training improved students’ overall course performance, completion of long-term tasks, and enhanced their self-satisfaction and perseverance [19]. In the same vein, Bell and Kozlowski’s [31] study aimed to identify the effect of adaptive guidance in enhancing self-regulation, knowledge, and performance in technology-based learning. The results revealed that adaptive guidance had a significant impact on the nature of trainees’ study and practice, self-regulation, and acquired knowledge and performance. Furthermore, adaptive mentoring led to significant improvements in the acquisition of basic knowledge and performance abilities early in training, and significant improvements in the acquisition of strategic knowledge and strategic performance skills later in training [31].

### 2.3. Studies That Have Examined the Impacts of Emotion and the Dual Model of Passion

Luxford et al. [32] employed path analysis to explore the correlation between harmonious and sympathetic cravings for video games, self-regulation, and overall well-being. Their findings revealed that individuals exhibiting higher levels of harmonious video game cravings demonstrated greater self-regulation, whereas those with elevated sympathetic cravings had lower self-regulation. Additionally, the study indicated that self-regulation played a more comprehensive role in explaining the interplay between emotion and well-being [32]. Similarly, Rihtarić, et al. [33] conducted a study to investigate the connection between harmonious and obsessive cravings for video games, school behavioral interaction, and their indirect relationship with school engagement through gaming time. Utilizing the binary emotion model, their pathway analysis unveiled direct effects of both harmonious and obsessive cravings on school behavioral reactivity, along with indirect effects through time spent playing video games. The results indicated that higher harmonious passion was linked both directly and indirectly to greater school engagement, albeit with some negative influence due to increased gaming time. Conversely, higher levels of obsessive passion were associated with reduced school involvement directly and indirectly due to prolonged gaming [33].

In contrast, Eckley et al. [34] focused on examining the interplay between psychological needs fulfillment, educational factors, and students’ expectations of future grades amidst the COVID-19 pandemic. Through structural equation modeling, they found that meeting higher needs significantly predicted engagement in educational factors, which in turn fostered resilience, motivation, and effective learning mechanisms. Positive mutual influences were identified between academics and students across various learning pathways. Reciprocal determinism emerged as the most intrinsically linked factor to predicted scores, shedding light on the interrelationships between emotion, trait conscientiousness, and self-regulated learning [34]. Similarly, Kaluge et al. [35] analyzed the adaptation of emotion measurement among Japanese literature students engaged in online learning in Indonesia. Their investigation considered five criteria—time, value, emotion, identity—along with student characteristics (gender and age) and attendance conditions (first-year vs. advanced year). Their findings unveiled differing passion dimensions, with first-year students showcasing higher harmonious passion and second-year students displaying greater obsessive passion [35].

Lastly, Yeh and Chu [36] aimed to categorize patterns of academic passion in e-learning by identifying four emotion types (internal and external harmonious passion, internal and external obsessive passion). The study explored connections between these emotions, self-regulation, and knowledge management in e-learning. Their structural equation modeling demonstrated interrelations among the four passion types, with learning-mediated self-regulation influencing the connection between academic passion and knowledge management. Notably, consensual passion held a more substantial role compared to compulsive passion in e-learning, influencing learning management among undergraduate students during the e-learning process [36].

The existing body of literature and research in this domain has primarily concentrated on investigating the reciprocal influence between passion and e-learning, as well as exploring the dynamics of self-regulation and knowledge management processes within distance learning environments. Additionally, these works have delved into examining the diverse impacts of emotions and the dual model of passion on distance learning experiences. On the basis of the preceding discussions, it can be deduced that the most noteworthy aspect of this particular study lies in its assessment of students’ learning emotions and the organization and management of knowledge, which can serve as indicators of the distance learning system’s quality [5,27,29,32,34,35,36]. Furthermore, this study sheds light on learners within distance learning contexts, encompassing their levels of satisfaction, patterns of interaction, and strategies for organizing and managing cognitive processes in digital environments. Moreover, the research examines the structural relationships between various variables, including the influence of learning emotions on knowledge management, mediated by self-regulation in the context of distance learning environments.

### 2.4. Theoretical Framework

A distance education system comprises various dimensions and elements that significantly contribute to its effectiveness, such as the learning environment, learning motives, passion for research beyond knowledge, learning organization, and learning management. Merely designing educational courses for e-learning and distance learning environments is no longer adequate to achieve desirable learning outcomes that satisfy students. Instead, it is crucial to consider learners’ motivation for learning. In this regard, Kim and Firck’s [37] study aimed to investigate the factors that stimulate and motivate students in e-learning from a distance, their level of motivation and perseverance, and how it changes during academic courses. The study concluded that several fundamental factors affect the level of motivation and learners’ continuity in learning effectively. Notably, perceived relevance, reported technology competence, and age emerged as the primary determinants of motivation to commence SDEL. Subsequently, during the SDEL process, perceived quality of instruction and learning (specifically, the belief that e-learning is suitable for one’s needs) and the initial motivation to begin were identified as the most influential factors in sustaining motivation. Moreover, the study established a noteworthy link between motivation during SDEL and positive changes in overall motivation. This, in turn, proved to be a significant predictor of learner satisfaction with SDEL. Consequently, the study’s findings shed light on essential instructional design principles that effectively sustain learner motivation in the realm of SDEL [37]. Furthermore, we will discuss the philosophical frameworks for the passion of learning and the essential differences between passion and motivation, as well as self-regulation and knowledge management. We will also examine the concept of the dual model of emotion and the structural relationship between emotion and its impact on knowledge management processes through the mediating role of self-regulation.

### 2.5. The Philosophical Frameworks for the Emotion of Learning and the Distinction between Passion and Motivation

According to Vallerand [38], passion refers to the driving force that motivates individuals to engage in a specific activity. It is the underlying motivation that inspires individuals to exhibit dedication, enthusiasm, and admiration towards an activity, concept, or person, and encourages them to invest regular time in it. From a philosophical perspective, passion is an essential human experience that provides individuals with the psychological energy to engage in activities that hold value for them. Without passion, individuals may struggle to find meaning in their lives [38]. Passion also plays a crucial role in enhancing individuals’ participation in the activities they are interested in, as highlighted by Curran Hill, Appleton [39,40].

Passion is often linked to motivation, curiosity, lifelong learning, self-efficacy, and a love of learning, but it is distinct from these concepts. Passion and self-efficacy are interconnected in a complex and reciprocal relationship [41]. Passion fuels motivation and perseverance, leading to mastery experiences that enhance self-efficacy. In turn, increased self-efficacy reinforces passion, resulting in continued commitment and achievement in the domain of interest. This interplay between passion and self-efficacy is instrumental in driving individuals towards success and personal fulfillment in their chosen pursuits. While motivation and passion share a close relationship [41], Vallerand [42] has sought to draw distinctions between the two concepts. Motivation serves as a theoretical construct encompassing internal and/or external influences that trigger, guide, intensify, and sustain behavior. According to this perspective, individuals are reactive entities responding to internal or external stimuli. In contrast, proponents of emotion theory perceive individuals as proactive beings who actively seek meaningful interactions with their environment to lead a purposeful life. Vallerand [42] posits “Passion necessitates a unique bond with an activity one holds dear. However, unlike intrinsic motivation, it mandates that the activity holds personal significance and contributes to one’s sense of identity to qualify as passion”. Consequently, a tennis player might be motivated to play due to their affection or fascination for the sport, yet their passion arises when tennis becomes an integral part of their identity [42].

Furthermore, passion assumes particular importance for a select few activities that propel us to thrive in life (e.g., a professional tennis player) due to its responsiveness, curiosity, enthusiasm, and unwavering commitment [43].

### 2.6. The Dual Model of Emotion in E-Learning Environments

Vallerand et al. [15] proposed a theoretical model known as the Dualistic Model of Passion to study individuals’ passion for activities. This model comprises two dimensions: harmonious passion, which arises from self-independence and willingness to engage in activities that align with the individual’s personality. It stems from a controlled inner feeling that allows the individual to freely and voluntarily pursue their passion activities without feeling pressured. This type of passion is characterized by a balanced integration with other aspects of an individual’s life, without causing any conflicts. On the other hand, obsessive passion results from planning a specific action that is subject to control, driven by an uncontrolled inner feeling that dominates the individual’s emotions when engaging in passionate activities on a repetitive and organized basis. This type of passion is characterized by internal pressures that compel the individual to engage in the activity, potentially leading to neglect of other essential activities, and causing conflicts between their passionate activity and other life domains [15].

Vallerand et al. [16] introduced innovative categories of emotion in the binary model of emotion, but did not provide clear indicators for harmonious craving, compulsive craving, or obsessive-compulsive disorder [15]. Previous studies have identified that internal or interpersonal factors such as curiosity, interest, and an internal need for self-improvement enhance an individual’s passion for learning. Conversely, external or interpersonal factors can significantly influence students’ eagerness in the context of e-learning [44,45]. Hence, in the context of e-learning, both harmonious and compulsive or obsessive cravings can be influenced by internal and external factors. Among the internal factors that influence harmonious craving, there are those that express controllable and harmonious emotions derived from enjoyment and satisfaction during interactions with others. Furthermore, external factors can influence harmonious craving, which expresses the harmonious and controllable emotion derived from self-designed enjoyment and satisfaction during interactions with others. In contrast, obsessive or compulsive craving is influenced by a group of internal factors, namely, excessive and uncontrollable cravings for an activity derived from internal forces such as impulsive and ego-centered thinking during interactions with the activity. Additionally, obsessive craving is affected by a group of external factors referring to excessive and uncontrollable passion towards an activity derived from external forces such as pressures and commitment during interactions with others [36].

It is worth noting that there are indicators of learners’ practices of harmonious passion in e-learning environments. For instance, learners tend to use e-learning to keep updated with the latest information, access rich educational materials and information that align with their competencies and meet their needs, and stimulate their ideas and learning through interactions with others. On the other hand, there are indicators of learners’ emotional obsessive practices in e-learning environments. For example, learners may feel uncomfortable when they fail to complete their e-learning requirements immediately after waking up, and they may find life boring if they do not use e-learning interfaces that day. They may also struggle to control their impulses to use e-learning, and usually use it only under the pressure of task deadlines. Additionally, they may feel obligated to use e-learning because their friends frequently learn through its interfaces [1].

### 2.7. Organizing and Managing Knowledge in E-Learning Environments

Self-regulation and knowledge management are essential steps and actions that learners can plan and adapt to organize and manage their learning effectively. These steps can help learners acquire information through their personal learning environment and smart assistant, ultimately leading to achieving their educational goals and improving their learning outcomes [46]. Self-regulation refers to a dynamic process through which learners construct their own learning activities by employing relevant cognitive and behavioral strategies. Within self-regulatory learning strategies, individuals utilize various techniques for acquiring knowledge, including actions such as organizing and transforming information, maintaining and overseeing records, and establishing learning objectives. A crucial aspect of self-regulation involves the capacity to monitor and control the learning processes themselves [47].

In contrast, knowledge management constitutes a systematic undertaking wherein learners actively generate knowledge through interactions with their environment. This process encompasses a range of competencies associated with acquiring and retaining knowledge, applying it in practical contexts, sharing knowledge with others, utilizing acquired knowledge effectively, incorporating new knowledge, and even generating original knowledge [7].

Indicators of learners’ practice of knowledge self-organization processes in e-learning environments include several factors, such as their ability to save essential information or links that help them identify the content they need, their capacity to choose e-learning interfaces that provide relevant information, their ability to adjust their search methods to obtain useful information, and their ability to adjust their criteria for choosing e-learning interfaces to find the ones that make them feel most comfortable when learning. Additionally, indicators of learners’ knowledge management practices in e-learning environments include obtaining information through search engines (such as Google and Yahoo), downloading static information (such as words and signs) from reviewed websites, and actively participating in e-learning communities (such as bulletin board systems, Facebook, etc.) to access important and current information.

### 2.8. The Structural Relationship of Emotion and Its Impact on Knowledge Management Processes through the Mediating Role of Self-Regulation in Distance Learning Environments (the Proposed Model)

The current study proposes a model that illustrates the structural relationship between emotion and its impact on knowledge management processes in distance learning environments, with the mediating role of self-regulation. The research proposes a hypothesis that suggests self-regulation might serve as an intermediary between emotion and knowledge management in the context of e-learning. Additionally, it postulates that passion could impact self-regulation within the realm of e-learning. However, it is important to note that passion alone may not suffice for achieving favorable learning outcomes unless learners effectively oversee their learning process. The self-regulation of learning encompasses three key components: the application of self-regulatory learning strategies, response to self-directed feedback regarding learning effectiveness, and interconnected motivational mechanisms. This framework is closely intertwined with the ability to establish learning objectives, as well as monitor and control the processes of learning [47].

Consequently, individuals who actively engage in self-regulation demonstrate a heightened involvement in their learning, taking proactive steps to seek out information as needed. This proactive approach nurtures self-awareness and a knowledgeable stance toward learning methods [8]. Therefore, self-regulation likely assumes a pivotal role as an intermediary factor that strengthens the impact of emotion on knowledge management within the e-learning milieu. Moreover, individuals exhibiting well-aligned passion may possess improved self-regulation skills, thereby facilitating effective knowledge management. Conversely, those harboring an affective obsession might impede self-regulation, potentially resulting in a mishandling of knowledge during the e-learning process.

**Figure 1:**
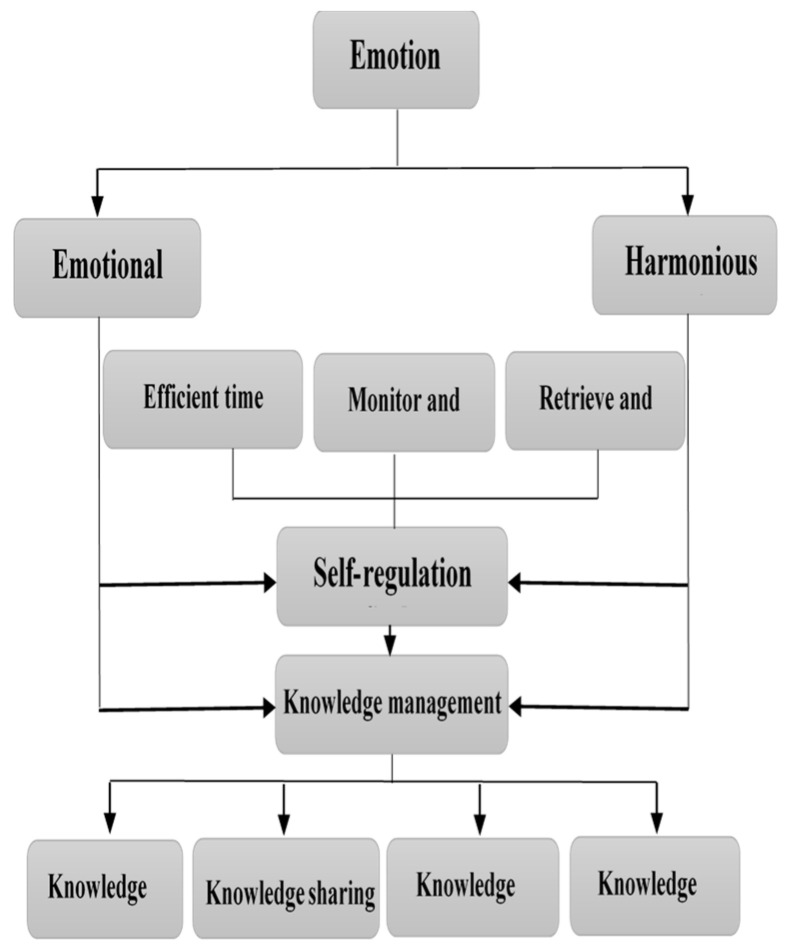
The proposed model of the structural relationships of emotion and its impact on knowledge management processes through the mediating role of self-regulation.

The proposed model of the structural relationship between emotion and its impact on knowledge management processes through the mediating role of self-regulation is depicted in Figure 1.

Figure 1 illustrates the proposed model of the structural relationship between emotion and its impact on knowledge management processes through the mediating role of self-regulation in distance learning environments, based on various indicators of the processes of learning emotion, organization, and knowledge management. The figure demonstrates that learning emotion, including harmonious passion and emotional obsession, plays a crucial role in influencing the processes of organizing and managing knowledge in distance learning environments. The proposed model assumes that self-regulatory processes, with their dimensions such as retrieving, organizing, controlling, scheduling, and efficient time management, will function as a mediator for the effects of emotion on knowledge management, and provide a distinctive contribution to explaining individual differences in the processes of acquisition, application, participation, formation, and knowledge management in a secure distance learning environment.

## 3. Materials and Methods

The current study relied on the descriptive correlational approach to identify the effects of the study variables (learning emotion: harmonious passion—emotional obsession) on knowledge management through the mediating role of self-regulation in distance learning environments.

### 3.1. Study Design

**Figure 2:**
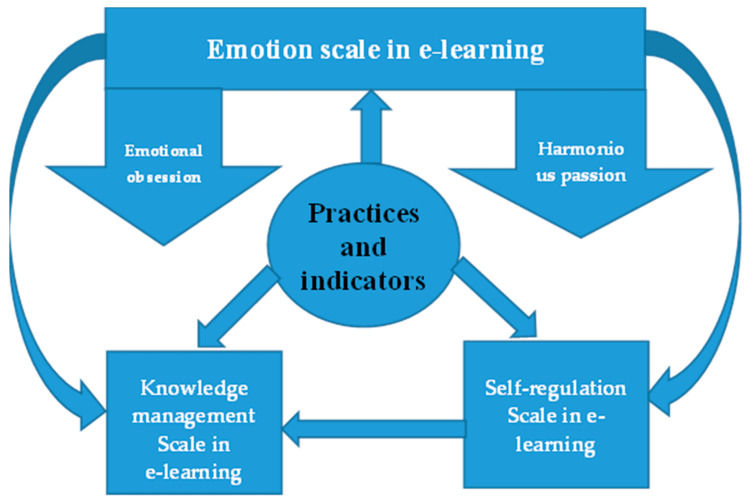
Learning emotion practices and their implications for the processes of organizing and managing knowledge in distance learning environments.

Figure 2 shows the design of the current research, where the research is based on identifying the degree of educational diploma students’ practice of the processes of learning emotion, management and organization of knowledge, and tracking the impact and reflections of learning emotion in its two parts, harmonious passion and emotional obsession, on the processes of organizing and managing knowledge in distance learning environments.

### 3.2. Population and Sample

The study’s population comprises a group of Saudi universities that offer e-learning and distance learning programs. Saudi universities are actively moving towards e-learning programs and integrating them into the Kingdom’s Vision 2030 plans. These universities provide all the necessary technological and technical capabilities to enable the e-learning process and develop specialized distance education programs that keep pace with international universities’ advancements. The researchers identified a group of Saudi universities that implement e-learning programs and randomly selected a sample of students enrolled in the distance education system during the academic year 2022/2023. The study sample consisted of 722 male and female students, with an average age falling within the range of 18 to 24 years.

### 3.3. Study Tools

In light of what the study aims to build tangible indicators of learning emotion processes (harmonious passion—emotional obsession) and the organization and management of knowledge in distance learning environments, the current study developed the following three scales: the emotion scale in distance learning, the scale of self-regulation in distance learning, and a scale of knowledge management in distance learning. The scales were prepared in the following steps:

Examination of previous studies and research related to the emotion of learning and its effects on the organization and management of knowledge in distance learning environments [5,32,33,34,36,38,48].

Constructing the initial copy of the scales: The emotion scale included two dimensions, the first dimension, harmonious passion, which included (12) items, and the second dimension, emotional obsession, which included (8) items. The scale of self-regulation in distance learning included the following three main dimensions: the first is retrieving and organizing information and includes (6) items, the second is strategy regulation and schedule monitoring and includes (7) items, and the third is time management efficiency and includes (3) items. The scale of knowledge management in distance learning included the following four main dimensions: the first is the acquisition and storage of knowledge and includes (7) items, the second is the application of knowledge and includes (6) items, the third is knowledge sharing and includes (5) items, and the fourth is the formation of knowledge and includes (4) items.

Determine the alternatives to the scales and their weights through the 4-point Likert scale as (strongly agree, agree, disagree and strongly disagree) with their weights. Weights (1, 2, 3 and 4) were determined, respectively, for each item.

#### 3.3.1. The Validation of the Reliability and Validity of the Scales

The scales were initially subjected to scrutiny by a panel of arbitrators, comprising faculty members holding the rank of professor in fields such as psychology, measurement, and evaluation, along with educational technology specialists hailing from various Saudi universities. Subsequent to their valuable feedback and suggestions, the researchers meticulously reviewed the question formulation and thoughtfully considered the proposed alternatives. As a result, necessary adjustments were made to the scales to ensure their refinement and appropriateness.

#### 3.3.2. Subsequently, a Pilot Study Involving 50 Students Was Conducted to Assess the Validity and Reliability of the Tools

Several indicators were examined to establish the construct validity of the measurement instrument, including Macdonald’s Omega and Composite Reliability. Additionally, the study assessed convergent and discriminant validity. To establish factor validity, confirmatory factor analysis (CFA) was employed, utilizing statistical software such as SPSS and Amos. CFA falls under the umbrella of structural equation modeling (SEM) and aims to uncover underlying patterns within data by analyzing relationships between latent constructs. This statistical methodology proves invaluable in various stages, including the creation of measurement tools, evaluation of construct validity, and analysis of methodological influences.

CFA serves as a cornerstone during the instrument’s development process, verifying the latent structure of the measurement tool. It also confirms the main dimensions and factor loadings inherent in the instrument. Thus, CFA emerges as an indispensable analytical technique, contributing significantly to other facets of psychometric assessment [2].

#### 3.3.3. Indicators and Coefficients of Construct Validity

In this section, we present the indicators and coefficients of construct validity. Macdonald’s Omega and composite reliability (CR) are commonly used to assess the reliability of scales. Table 1 displays the results of these indicators, revealing that the values of Macdonald’s Omega and CR range from 0.897 to 0.956 and 0.899 to 0.959, respectively. These values are consistent with the recommended values (>0.7), indicating substantial internal consistency of the scales. Additionally, the values of average variance extracted (AVE) range from 0.673 to 0.795, exceeding the minimum threshold of 50%. Furthermore, the discriminant validity coefficients (square root of the AVE) must be greater than the inter-correlations between the latent variables or factors. By comparing the last column, we find that this criterion is met, as it exceeds the minimum loading factor value. These outcomes demonstrate that the scales are both reliable and valid [49].

#### 3.3.4. Construct Validity according to Confirmatory Factor Analysis (CFA)

To guarantee the factorial construct validity, the study sample was administered the final version of the scale, and a confirmatory factor analysis was performed to evaluate the extent to which the scale items fit within their respective dimensions.

**Figure 3:**
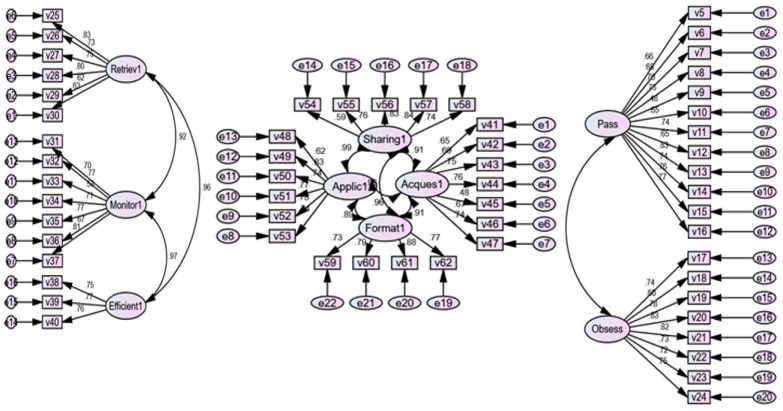
The results of the confirmatory factor analysis to establish the relationship between the scale items and their respective dimensions and degree of loading.

To develop the scale, it was crucial to determine the loading values of the scale items on their respective dimensions, as illustrated in Figure 3. The loading factors of the items needed to meet the criterion that items with loading factors less than 0.40 should not be adopted [50]. The results indicate that all items have loading factors greater than 0.40 within their respective dimensions.

## 4. Results

To address the first research question, which sought to determine the extent of students’ practice of learning emotion processes, knowledge management, and organization in distance learning environments, arithmetic means, standard deviation, rank determination, and level of practice were utilized. Each of the three dimensions of the scales, as well as the scales as a whole, were evaluated, as presented in Table 2.

Table 2reveals that the arithmetic mean of the items on the emotion scale was 2.9085, indicating that the level of students’ practice of learning emotion processes in distance learning environments was above average. The students’ practice of “harmonious passion” was higher than “emotional obsession”, as indicated by an arithmetic mean of 3.1069. The self-regulation scale had an arithmetic mean of 3.1731, suggesting that the level of students’ practice of self-regulation of knowledge in distance learning environments was higher than average. The knowledge retrieval and organization dimension received a high score, with an arithmetic mean of 3.2759, indicating that students practice knowledge retrieval and organization more effectively than other dimensions in distance learning environments. Lastly, the arithmetic mean of the items on the knowledge management scale was 3.0947, indicating that the level of students’ practice of knowledge management processes in distance learning environments was higher than average. The knowledge acquisition dimension obtained a high score, with an arithmetic mean of 3.1783, implying that students practice knowledge acquisition processes more proficiently than other dimensions in distance learning environments.

To address the second research question, which aimed to determine the statistical significance at a level of 0.05 of students’ responses to the emotion, self-regulation, and knowledge management scales in relation to the variables of gender, specialization, level of computer use, and computer courses acquired, various statistical measures were employed. Arithmetic means, standard deviations, and t-values were calculated to indicate any statistical differences between the averages of students’ responses on the emotion, self-regulation, and knowledge management scales with respect to the variables of gender and specialization [51]. The results of these analyses are presented in Table 3.

Table 3 reveals that there are no statistically significant differences between the averages of students’ responses to the emotion, self-regulation, and knowledge management scales based on gender and specialization.

A three-way ANOVA was employed to determine the statistical significance of students’ responses to the emotion, self-regulation, and knowledge management scales in relation to the level of computer use and computer courses acquired, as shown in the Table 4.

Table 4 indicates that there are statistically significant differences at α ≤ 0.05, between the averages of students’ responses to the emotion, self-regulation, and knowledge management scales according to the level of computer use in favor of excellent levels. It also shows that there are statistically significant differences at the level of α ≤ 0.05 between the averages of students’ responses to the scale of emotion, self-regulation, and knowledge management according to the variables of acquired computer courses in favor of advanced levels.

To address the third research question, which aimed to investigate the relationship between learning emotion (harmonious passion and emotional obsession) and self-regulation and knowledge management in distance learning environments, the Pearson correlation coefficient was computed. This coefficient was calculated for students’ responses to the emotion scale and between the two scales of self-regulation and knowledge management, as shown in Table 5.

Table 5 reveals that there is a significant correlation between the emotion scale (passion and obsession) and both self-regulation and knowledge management in distance learning environments. The correlation coefficient between the emotion scale and both self-regulation and knowledge management was found to be 0.722 and 0.694, respectively. Additionally, the correlation coefficient between harmonious passion and both self-regulation and knowledge management was 0.709 and 0.716, respectively, while the correlation coefficient between emotional obsession and both self-regulation and knowledge management was 0.162 and 0.239, respectively. Furthermore, it is evident that the association of harmonious passion with both the processes of self-regulation and knowledge management is stronger than the association of emotional obsession with both the processes of self-regulation and knowledge management in distance learning environments.

**Figure 4:**
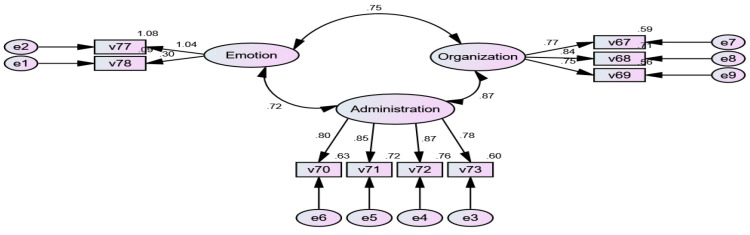
The accuracy and loading factors of dimensions within the scales.

To ensure the accuracy and validity of the representation of the items on each of the three scales, as well as to measure the extent of the correlation among these scales, the AMOS program was utilized. This program is a robust and reliable tool for calculating the accuracy and validity of items. Figure 4 displays the loading of each dimension of the scale in the overall scale, along with the correlation coefficient between the scales based on the accuracy, validity, and loading of each dimension for each scale.

Figure 4 indicates that the dimension of harmonious passion in the emotion scale had a loading degree of approximately 1.08, while the dimension of emotional obsession in the emotion scale had a loading degree of 1.04. The following dimensions had loading degrees in the knowledge management scale: acquiring knowledge (0.80), applying knowledge (0.85), knowledge sharing (0.87), and knowledge formation (0.78). Additionally, the knowledge organization scale had the following dimensions with loading degrees: retrieving and organizing (0.77), monitoring and scheduling (0.84), and time management (0.84). According to the AMOS program, the correlation coefficients were calculated among the three scales. The correlation coefficient between the emotion scale and knowledge organization was (0.75), the correlation coefficient between emotion and knowledge management was (0.72), and the correlation coefficient between knowledge organization and knowledge management was (0.87). These results confirm the existence of a significant correlation between the emotion, self-regulation, and knowledge management scales.

To address the fourth research question, which aimed to investigate the effect of learning emotion (harmonious passion and emotional obsession) on self-regulation and knowledge management, a two-way analysis of variance was conducted. The results of this analysis reveal the following:

Table 6 reveals that learning emotion (harmonious passion and emotional obsession) has a statistically significant impact on both self-regulation and knowledge management. These findings confirm that learning emotion practices, as represented by harmonious passion and emotional obsession, play a significant role in influencing the processes of organizing and managing knowledge in distance learning environments.

To test the primary hypothesis of this study, which posits that “harmonious passion and obsessive passion make distinct contributions to explaining individual differences in knowledge management, through the mediating role of self-regulation in distance learning environments”, the following was conducted.

First, to test the validity of the proposed pathway model and examine the relationships between harmonious passion (v77), emotional obsession (v78), self-regulation (v75), and knowledge management in distance learning environments (v76), structural equation modeling (SEM) was conducted using the AMOS program. Figure 4 illustrates the proposed model.

**Figure 5:**
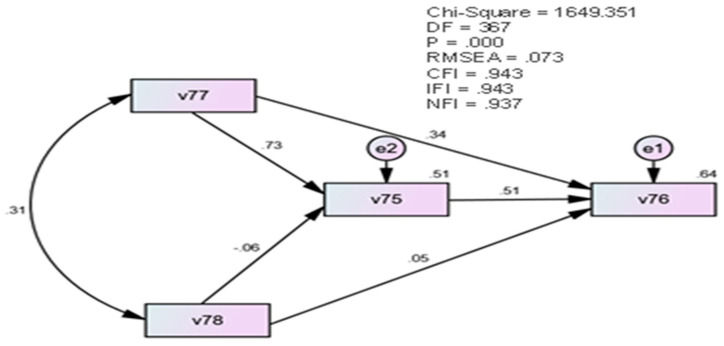
The proposed path model for the study using structural equation modeling (SEM).

Figure 5 illustrates that harmonious passion has a direct impact on knowledge management, with an effect rate of (0.34), while emotional obsession has a direct impact on knowledge management, with an effect rate of (0.05). These findings suggest that the direct impact of harmonious passion on knowledge management is significantly greater than the effect of emotional obsession on knowledge management. Furthermore, the proposed model indicates that self-regulation has a direct impact on knowledge management, with an effect rate of (0.51). Furthermore, Figure 4 reveals that there is an indirect effect of harmonious passion on knowledge management through the mediating role of self-regulation, with a direct effect of (0.73). Additionally, there is an indirect effect of emotional obsession on knowledge management through the mediating role of self-regulation, with a direct effect ratio of (−0.06). These results suggest that the effects of harmonious passion on knowledge management through the mediating role of self-regulation were positive and strong, whereas the effects of emotional obsession on knowledge management through the mediating role of self-regulation were negative and weak.

To assess the quality of the proposed model, which examined the effects of learning emotion on knowledge management through the mediating role of self-regulation in distance learning environments, the AMOS program was utilized. The following fitness indices were examined to determine the quality of the proposed model: Comparative Fit Index (CFI), Normative Fit Index (NFI), and Incremental Fit Index (IFI). Acceptable values for these indices are greater than 0.90. Additionally, the root mean square error of the approximation (RMSEA) should be less than 0.08, and the *p* value should be greater than 0.05 for the model to be acceptable [1]. Table 7 illustrates that the proposed model conforms well to the data, as per the assumed model, based on these indices.

Table 7 demonstrates that all indicators of the confirmatory factor analysis results suggest the quality of the proposed model, and that the proposed model matches the assumed model for the sample data to a large extent. Thus, the main hypothesis of the study can be accepted, which is that harmonious passion and obsessive passion have a significant influence on individual differences in knowledge management, mediated by self-regulation in distance learning environments.

## 5. Discussion

This study’s findings demonstrate that the distance learning practices related to learning emotion processes, knowledge management, and organization were higher than average among the study sample, indicating the students’ behavioral practices in the distance learning system concerning harmonious passion and emotional obsession. Additionally, the practices of knowledge acquisition, application, sharing, formation, retrieval, organizing, control, and scheduling, as well as efficient time management, were acceptable. This can be attributed to the developed system specialized in e-learning and distance education by Saudi universities, which follows international standards. The e-learning system includes e-learning management systems and synchronous and asynchronous education systems, meeting the increasing demand of students for education through the distance learning system to obtain professional certificates. This responsibility has naturally increased students’ desire to delve deeper into their fields of study, raising their passion for research and access to knowledge and skills related to their disciplines. These findings are consistent with Al Ali and Saleh’s study [1].

This study’s findings indicate no statistically significant differences between the mean scores of students’ responses on the emotion, self-regulation, and knowledge management scales based on gender and specialization variables. This can be attributed to the widespread culture of distance education and equal provision of educational services for both genders, leading to equal opportunities for motivation and passion for searching beyond knowledge. Furthermore, both literary and scientific disciplines offer educational paths that satisfy the learners’ desires and provide equal employment opportunities. However, the study revealed statistically significant differences between the mean scores of students’ responses on the emotion, self-regulation, and knowledge management scales based on the level of computer usage, favoring excellent levels, and the acquired computer courses, favoring advanced levels. These findings can be explained by the fact that obtaining advanced levels in computer courses positively affects learners’ ability to use and employ computers for education and distance learning purposes.

This study’s findings also reveal that the emotion scale’s two dimensions, harmonious passion and emotional obsession, and the knowledge management scale’s dimensions of knowledge acquisition, application, sharing, formation, retrieval, organization, control, and scheduling, as well as efficient time management, have a significant loading degree. This confirms the accuracy and validity of each scale’s expressions and dimensions (factor loading), which significantly contributed to the correlation between the emotion, self-regulation, and knowledge management measures. These findings are consistent with Yeh’s study [5].

This study’s results reveal a statistically significant impact of learning emotion, encompassing both harmonious passion and emotional obsession, on the processes of self-regulation and knowledge management. Within this context, multimedia motivation emerges as an internal state that triggers, sustains, and propels learners to actively participate in their learning endeavors. Passion, on the other hand, is linked to elevated levels of learning comprehension [52] and serves as a driving force for learners to fully immerse themselves in their educational pursuits, thus facilitating their persistence and goal attainment [53].

Affective craving pertains to a robust and independent inclination to engage in an activity under one’s control, allowing for adaptability in involvement. In contrast, affective obsession involves a potent urge to engage in an activity as if the activity exerts control over the individual [52,54]. As a result, university students exhibiting heightened levels of harmonious passion are better positioned to manage, organize, and direct their behavior in e-learning settings. Consequently, in the context of distance learning environments, harmonious passion is expected to wield greater influence than emotional obsession.

The primary objective of this study was to develop tangible indicators for measuring emotion in distance learning environments by constructing a binary model of emotion. This model aimed to investigate the relationships between harmonious passion, emotional obsession, self-regulation, and knowledge management in distance learning environments. To achieve this objective, a two-dimensional model of emotion was proposed to explain the structural relationship of emotion and its impact on knowledge management processes through the mediating role of self-regulation. The proposed model aimed to ensure that harmonious passion and emotional obsession contribute uniquely to explaining individual differences in knowledge management through the mediating role of self-regulation in distance learning environments. After examining the proposed pathway model using SEM, the results reveal that sympathetic craving is a more significant predictor of self-regulation and knowledge management than emotional obsession. Furthermore, although emotion (harmonious passion and emotional obsession) is correlated and predictive of self-regulation and knowledge management in e-learning, harmonious passion and emotional obsession have direct and indirect effects in opposite directions. Specifically, harmonious passion positively influenced self-regulation and knowledge management, while emotional obsession had a negative impact on self-regulation and knowledge management. These findings are consistent with Philippe et al.’s study [55].

The SEM analysis results in this study confirm the validity of the proposed pathway model, indicating that self-regulation plays a crucial mediating role between emotion and knowledge management. In particular, college students characterized by harmonious passion are predisposed to engage in self-regulated learning practices, which in turn foster effective knowledge management. Conversely, the presence of obsessive emotion is likely to impede self-regulation and hinder the efficient management of knowledge. Within the pervasive e-learning landscape, learners encounter a vast array of learning resources, necessitating adept knowledge management to streamline their learning journeys [56].

Knowledge management serves to expedite students’ learning processes by tailoring them to their individual preferences. Proficient self-regulatory learners exhibit heightened awareness of their learning strategies, actively monitor their learning processes, and adeptly modify their goals. This concerted effort maximizes their overall endeavors and consequent success [57,58,59]. Consequently, learners endowed with robust self-regulatory skills prove adept at acquiring, retaining, applying, sharing, and even generating knowledge within e-learning contexts.

These findings align with previous studies conducted by Gillet et al. [60] and Sheldon and Elliot [61], underscoring the essential role of self-regulation in effective knowledge management within the realm of e-learning.

## 6. Conclusions

The widespread adoption and expansion of distance learning highlights the need to address issues related to learning motivation, research passion, learning organization, and management, which are essential factors that significantly impact the success of the distance learning system. Thus, it is crucial to explore effective ways to utilize knowledge management in e-learning environments, which are closely related to the individual and their learning methods. To achieve this, it is necessary to establish measurable indicators for the various dimensions of knowledge management processes to assess the degree of learners’ engagement in learning emotion processes and knowledge management and organization in e-learning environments.

Due to the scarcity of studies that explore the relationship between self-regulation and knowledge management in e-learning environments, this study contributes to the literature by presenting a two-dimensional model of emotion. The study confirms that while there is a positive correlation between harmonious passion and emotional obsession, they interact in ways that have varying effects on self-organization and knowledge management in e-learning environments. This sheds light on the need for educational designers and developers in the digital society to consider designing activities in e-learning environments that promote harmonious passion while reducing emotional obsession. Moreover, it is crucial to transform practices related to obsessive-compulsive processes to practices associated with harmonious passion processes to enhance university students’ self-regulation and knowledge management abilities. This can be achieved by incorporating teaching strategies that provide a high-quality interactive Internet environment with features such as promoting self-directed learning, encouraging discussions, arousing curiosity and interest through different learning tasks, encouraging independent learning, and providing immediate feedback.

Despite the relatively large sample size of this study, it is important to note that it is a survey, and further applied studies are necessary to test the proposed pathway model and validate the findings with different samples to enhance the study’s credibility and validity. Based on the study’s results, the researchers offer several recommendations and proposals, including training and encouraging faculty members to create a high-quality interactive Internet environment by employing various teaching strategies to strengthen the feelings of harmonious passion and reduce emotional obsession. Moreover, conducting quasi-experimental research based on various extension approaches is necessary to develop harmonious passion, organization, and knowledge management among distance learners.

## Figures and Tables

**Table 1:** Several indicators and coefficients used to assess construct validity.

Scale	Constructs	Items	Loading Factor	Macdonald’s Omega	CR	AVE	$\sqrt{AVE}$
knowledge management	Knowledge acquisition and storage	7	0.45–0.67	0.872	0.872	0.673	0.820
	Knowledge application	6	0.62–0.66	0.878	0.877	0.765	0.875
	Knowledge sharing	5	0.58–0.68	0.873	0.871	0.795	0.892
	knowledge formation	4	0.52–0.69	0.907	0.907	0.770	0.877
	Retrieving and organizing information	6	0.43–0.47	0.841	0.843	0.723	0.850
Knowledge self-organization	Strategy regulation and schedule monitoring	7	0.42–0.58	0.910	0.912	0.734	0.857
	Efficient time management	3	0.41–0.52	0.886	0.886	0.783	0.885
passion	Harmonious passion	21	0.51–0.68	0.95	0.95	0.718	0.847
	emotional obsession	8	0.50–0.64	0.72	0.72	0.773	0.879

**Table 2:** The means, standard deviation, practice level, and rank.

Scale	Dimension	Mean	Standard Deviation	Practice Level	Rank
Emotional	Harmonious passion	3.1069	0.38773	above average	1
	emotional obsession	2.7101	0.47598	above average	2
Whole scale		2.9085	0.35054		
Knowledge self-organization	Retrieving and organizing information	3.2759	0.37772	average	1
	Strategy regulation and schedule monitoring	3.0830	0.45150	above average	3
	Efficient time management	3.1605	0.45511	above average	2
Whole scale		3.1731	0.36944	above average	
knowledge management	Knowledge acquisition and storage	3.1783	0.44338	above average	1
	Knowledge application	3.0916	0.42074	above average	3
	Knowledge sharing	2.9938	0.48526	above average	4
	knowledge formation	3.1149	0.47401	above average	2
Whole scale		3.0947	0.39638	above average	

**Table 3:** The means, standard deviations, and t-values for students’ responses on the emotion, self-regulation, and knowledge management scales, according to the gender variable.

Scale	Gender	N	Mean	*t*-Value	Sig.
Emotional	Male	252	2.9507	0.40298	0.519
	Female	470	2.8991	0.33781	
Knowledge self-organization	Male	252	3.2100	0.41759	0.121
	Female	470	3.1648	0.35810	
knowledge management	Male	252	3.0896	0.48172	0.424
	Female	470	3.0958	0.37566	
Emotional	humanity	389	2.9051	0.35219	0.519
	scientific	333	2.9134	0.34947	
Knowledge self-organization	humanity	389	3.1577	0.35800	0.644
	scientific	333	3.1950	0.38541	
knowledge management	humanity	389	3.0656	0.41799	0.540
	scientific	333	3.1360	0.36099	

**Table 4:** Results of three-way ANOVA of the averages of students’ responses to the emotion, self-regulation, and knowledge management scales according to the level of computer use and computer courses acquired.

		Mean	Sum of Squares	df	Mean Square	F	Sig.
Emotional	weak	2.9283	1.181	3	0.394	3.273	0.021
	Medium	2.8398					
	good	2.8972					
	Excellent	3.0345					
Knowledge self-organization	weak	2.9790	5.290	3	1.763	14.556	0.000
	Medium	3.0497					
	good	3.1781					
	Excellent	3.4255					
knowledge management	weak	2.9689	3.943	3	1.314	8.989	0.000
	Medium	2.9872					
	good	3.0896					
	Excellent	3.3252					
Emotional	No courses	2.8788	2.832	2	1.416	12.337	0.000
	Basic level	2.8584					
	advanced level	3.1212					
Knowledge self-organization	No courses	3.0792	3.306	2	1.653	13.020	0.000
	Medium	3.1778					
	good	3.3800					
knowledge management	No courses	3.0404	3.053	2	1.526	10.276	0.000
	Medium	3.0618					
	good	3.3154					

**Table 5:** Pearson correlation coefficient for students’ responses to the emotion scale and between the two scales of self-regulation and knowledge management.

	Emotional	Passion	Obsession	Organization	Management
Emotional	-				
Passion	0.764 **	-			
Obsession	0.851 **	0.311 **	-		
organization	0.722 **	0.709 **	0.162 **	-	
Management	0.694 **	0.716 **	0.239 **	0.758 **	-

** Correlation is significant at the 0.01 level (2-tailed).

**Table 6:** The results of a two-way analysis of variance conducted to examine the impact of learning emotion (harmonious passion and emotional obsession) on self-regulation and knowledge management.

		Sum of Squares	df	Mean Square	F	Sig
self-regulation	Emotion	22.731	115	0.198	2.436	0.000
	Passion	33.488	115	0.291	4.061	0.000
	Obsession	33.288	115	0.289	1.512	0.005
knowledge management	Emotion	22.731	115	0.198	2.436	0.000
	Passion	33.488	115	0.291	4.061	0.000
	Obsession	33.288	115	0.289	1.512	0.005

**Table 7:** The quality indicators of the proposed model, which examined the effects of learning emotion on knowledge management through the mediating role of self-regulation in distance learning environments.

Name of Category	Indicators of the Internal Construct Validity	Level of Acceptance	Indexes in the Proposed Model
Absolute fit	ChiSq	*p* > 0.05	Significant
	RMSE	RMSE < 0.08	0.073
Incremental fit	CFI	CFI > 0.90	0.943
	TLI	TLI > 0.90	0.943
	NFI	NFI > 0.90	0.937
Parsimonious fit	Chisq/df	Chis/df < 5.0	Chisq/df = 4.49 < 5.0

## Data Availability

Data supporting the findings and conclusions are available upon request from the corresponding author.
